# Access to High-Cost Medications After a Cap on Monthly Out-of-Pocket Spending in California

**DOI:** 10.1001/jamanetworkopen.2021.26642

**Published:** 2021-09-24

**Authors:** Erin Audrey Taylor, Christine Buttorff, Vishaal Pegany, Lindsay Petersen, Allison Mangiaracino, Emory Wolf, Lance Lang

**Affiliations:** 1 RAND Corporation, Santa Monica, California; 2 RAND Corporation, Arlington, Virginia; 3 California Health and Human Services Agency, Sacramento; 4 San Francisco Health Plan, San Francisco, California; 5 Health Plan Regulatory and Exchange Operations, Kaiser Permanente, Sacramento, California; 6 Covered California, Sacramento

## Abstract

This cross-sectional study evaluates the association of a cap on monthly out-of-pocket spending on high-cost drugs with use of these drugs and out-of-pocket spending in California in 2015 and 2016.

## Introduction

High prescription drug costs are a substantial problem for many patients who cannot afford their medications, even with health insurance. Capping monthly out-of-pocket (OOP) costs for high-cost drugs may increase the affordability of these medications. In January 2016, Covered California implemented a monthly cap on OOP costs for high-cost drugs to increase patient access. We evaluated the association of this cap with high-cost drug use and OOP spending.

## Methods

This cross-sectional study used pharmacy claims data from private insurers offering coverage in Covered California (California’s Patient and Protection and Affordable Care Act marketplace) from 2015 to 2016. These data represent 9 of the 11 participating insurers, covering 91% (2016) to 93% (2015) of enrollees with drug coverage. We defined cap-eligible drugs as those with a proxy price (imputed price for insurers not reporting cost information) greater than the 30-day supply threshold cost for each metal tier in 2015 or 2016 (details are given in the eMethods in the Supplement). The RAND Human Subjects Protection Committee exempted this research from human participants protection because it used secondary data. This study followed the Strengthening the Reporting of Observational Studies in Epidemiology (STROBE) reporting guideline.

We identified patients continuously enrolled during 2015 and 2016 with at least 1 cap-eligible prescription drug fill in either year. We measured adherence as the total annual days supplied per patient. Because patients may take multiple medications, we also analyzed the month in which patients reached their annual maximum OOP spending (if they reached it). Results are stratified by metal tier, which is the marketplace indication of plan generosity, with Bronze being the least generous and Platinum being the most generous. Metal tiers have different cost-sharing caps, and sicker patients may enroll in metal tiers with more generous coverage. Data were analyzed using Microsoft Excel and Access. Analyses were performed from May 1, 2019, through June 30, 2020.

## Results

**Figure 1.**
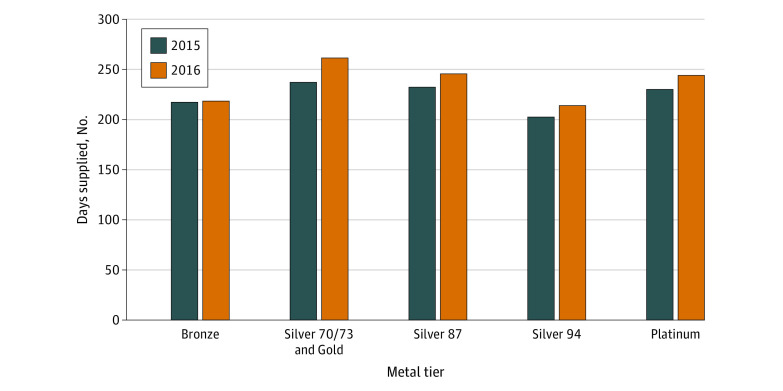
Mean Annual Days Supplied for Cap-Eligible Drugs From 2015 to 2016 Data are from Covered California claims from 2015 to 2016.

The number of patients who filled at least 1 cap-eligible drug increased from 2015 to 2016, with 1442 and 1927 Silver enrollees filling a cap-eligible drug in 2015 and 2016, respectively. We found no change in the total days supplied for patients in Bronze plans but small increases in the mean days supplied for the other metal tiers (Figure 1).

**Figure 2.**
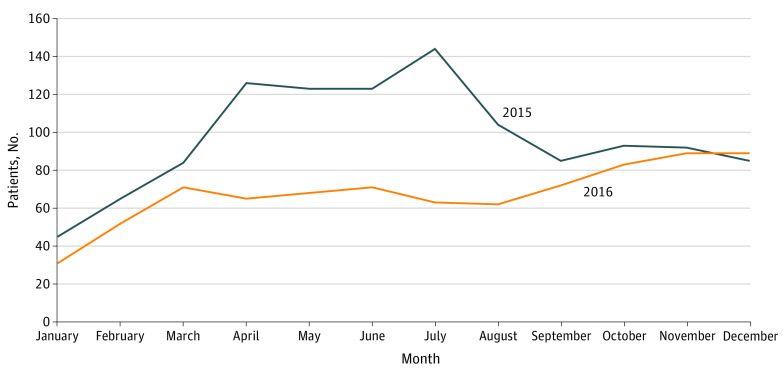
Number of Patients Reaching the Maximum Out-of-Pocket Limit for Their Plan by Month Data are from Covered California claims from 2015 to 2016.

Fewer patients reached the maximum OOP spending in each month in 2016 than in 2015 (Figure 2). Of the 4255 patients in 2015 who filled at least 1 cap-eligible prescription, 1169 (27.4%) reached the maximum OOP spending. In 2016, 816 of 5165 patients (15.8%) reached the maximum. Although the number of patients reaching the maximum OOP spending was similar in the early part of each year, a larger number of patients reached the maximum in the middle of the year in 2015, and the number of patients reaching the maximum in 2016 was higher toward the end of the year.

## Discussion

The findings of this cross-sectional study suggest that Covered California’s drug cap policy was associated with modest changes in high-cost drug use and adherence and with making expenses more consistent throughout the year. Although these results were found for Silver, Gold, and Platinum plans, they were not found for Bronze plans. Fewer enrollees reached the annual maximum OOP spending after implementation of the policy.

This study has limitations. This was a descriptive study; more detailed statistical analyses will be needed to establish causation. The proxy costs used to identify cap-eligible drugs are estimates and may not reflect actual spending. Similar to proxy costs, plan sponsors varied in the extent they reported cost-sharing amounts, although not over time. Thus, our results likely represent an undercount of the number of patients reaching the annual maximum OOP spending.

Caps on prescription drug OOP spending are relatively new. Most states that have enacted caps have focused on chemotherapy medications,^1^ which have slightly lowered OOP costs for patients.^2^ Other states have implemented or are considering similar policies for other drugs,^3,4^ such as insulin.^5,6^ Our findings suggest that cost-sharing caps for high-cost drugs may be associated with increased adherence and increased consistency of OOP spending for patients.

## Notes

Supplement.
eMethods
