# Supplementary material for: Access to High-Cost Medications After a Cap on Monthly Out-of-Pocket Spending in California
Source: JAMA Netw Open. 2021 Sep 24;4(9):e2126642. doi: 10.1001/jamanetworkopen.2021.26642 (PMC8463936; doi:10.1001/jamanetworkopen.2021.26642)
Supplement: Supplement. — eMethods [file jamanetwopen-e2126642-s001.pdf]

Supplemental Online Content

Taylor EA, Buttorff C, Pegany V, et al. Access to high-cost medications after a cap on monthly out-of-pocket spending in California. *JAMA Netw Open.* 2021;4(9):e2126642. doi:10.1001/jamanetworkopen.2021.26642

eMethods.

This supplemental material has been provided by the authors to give readers additional information about their work.

© 2021 Taylor EA et al. *JAMA Network Open.*

eMethods.

On January 1, 2016, California's ACA Marketplace (Covered California) implemented upper limits on out-of-pocket costs for expensive drugs over a certain dollar value that depended on the metal tier. The ACA organized the generosity, or actuarial value, of plans into four metal tiers, with bronze being the least and platinum being the most generous. The maximum patient out-of-pocket cost for a 30-day supply differs by metal tier, ranging from $150 for some Silver plans to $500 for Bronze plans under the new policy.

We analyzed a new data source of pharmacy claims data from private insurers offering coverage in the Covered California individual health insurance market. These data represent all pharmacy claims for nine of the eleven participating issuers; 91% (2016) to 93% (2015) of Covered California enrollees eligible for prescription drug coverage have claims included in the data. Pharmacy claims include the National Drug Code (NDC) used to identify the drug, date filled, days supplied, quantity supplied, and drug name. We identified drugs eligible for the cap by looking at claims data for 2015 and 2016, the years prior to and just after the implementation of the cap. We looked for any drug with a proxy price greater than the 30-day normalized supply threshold cost for each metal tier. (Drugs eligible for the cap are listed at the end of this file.) The proxy price and out-of-pocket spending amounts were calculated by IBM Watson, which manages these data through a contract with Covered CA. Not all insurers submit spending data (e.g. the copayments and allowed amounts) so IBM Watson imputes spending with a proprietary algorithm. Our estimates of out-of-pocket spending are based on plan-provided data where the data quality (e.g., missingness, accuracy) varies across different plan sponsors. We analyzed data for the years 2015 and 2016 (the year before and the first year after implementation of the cost sharing cap).

© 2021 Taylor EA et al. *JAMA Network Open.*

We included patients continuously enrolled in the same Covered California plan for both 2015 and 2016, in order to ensure that we are able to capture their complete annual prescription drug utilization history. We further restrict the sample to patients who filled at least one cap-eligible drug in one or both of those years so as to characterize any changes in days supplied for those patients who had at least one fill.

We measured utilization as the number of 30-day fills of cap-eligible drugs per patient. Adherence was measured as the total annual days supplied per patient. Finally, we captured the month in which patients hit their out-of-pocket maximum, if they reached the maximum in the year.

For each of the outcomes described above, we generated descriptive statistics. We stratify our results by the metal tier, as different metal tiers have different cost sharing caps and eligibility rules as noted above. Furthermore, there can be unobserved selection into metal tier based on health status (less healthy individuals may seek more generous plans) so we analyze the utilization by tier.

There are some limitations to this analysis. First, this is a descriptive study intended to provide an initial assessment of the effect of the cost sharing cap on utilization and costs. More detailed statistical analyses controlling for other factors that might contribute to prescription drug utilization and spending will need to be conducted to determine whether the findings shown here can be attributed to the new policy. Second, the Covered California data do not contain actual incurred costs for many of the participating insurers. This means that the proxy costs used to construct the set of cap-eligible drugs may not reflect actual spending. These costs came from Marketscan data; we were not able to assess differences between actual plan costs and the

© 2021 Taylor EA et al. *JAMA Network Open.*

Marketscan proxy price. Finally, other changes to formulary requirements occurred in 2016 and may contribute to these findings.

List of Drugs Eligible for the Cost Sharing Cap in 2015 and/or 2016

**Product Name**
ABACAVIR SULFATE, LAMIVUDINE AND ZIDOVUDINE
ABACAVIR SULFATE-LAMIVUDINE
ABRAXANE
ACTEMRA
ACTHAR
ADCIRCA
ADEMPAS
ADVATE
AFINITOR
AKYNZEO
ALECENSA
AMICAR
AMPHOTERICIN B
AMPYRA
APTIVUS
ARANESP
ARISTADA
ATRIPLA
AUBAGIO
AVONEX
AVONEX PEN
BARACLUDE
BENEFIX
BENLYSTA
BETASERON
BEXAROTENE
BOSULIF
BUNAVAIL
BUPRENORPHINE-NALOXONE
CABOMETYX
CAPECITABINE
CAPRELSA

© 2021 Taylor EA et al. *JAMA Network Open.*

CAYSTON
CELLCEPT
CHORIONIC GONADOTROPIN
CIMZIA
CINRYZE
COMETRIQ
COMPLERA
COPAXONE
COSENTYX
COTELLIC
CUPRIMINE
CYCLOPHOSPHAMIDE
CYCLOSPORINE
DAKLINZA
DEPEN
DESCOVY
EDURANT
EGRIFTA
ENBREL
ENOXAPARIN SODIUM
ENTECAVIR
ENTERAGAM
ENTYVIO
ENVARSUS XR
EPCLUSA
EPZICOM
ERIVEDGE
ESBRIET
ETOPOSIDE
EVOTAZ
EXJADE
EXTAVIA
FIRAZYR
FONDAPARINUX SODIUM
FORTEO
FRAGMIN
FUZEON
GAMMAGARD LIQUID
GAMUNEX-C

© 2021 Taylor EA et al. *JAMA Network Open.*

GEMCITABINE
GENOTROPIN MINIQUICK
GENVOYA
GILENYA
GILOTRIF
GLATOPA
GLEEVEC
GONAL-F RFF REDI-JECT
GRANIX
HARVONI
HIZENTRA
HUMATROPE
HUMIRA
HYCAMTIN
IBRANCE
ICLUSIG
IDELVION
IMATINIB MESYLATE
IMBRUVICA
INLYTA
INTELENCE
INTRON A
INVEGA SUSTENNA
INVEGA TRINZA
IRESSA
ISENTRESS
JADENU
JAKAFI
JUXTAPID
KALETRA
KALYDECO
KINERET
KRYSTEXXA
KUVAN
LENVIMA
LETAIRIS
LEUKERAN
LEUKINE
LEXIVA

© 2021 Taylor EA et al. *JAMA Network Open.*

LONSURF
LOVENOX
LUCENTIS
LUPRON DEPOT
LYNPARZA
MAKENA
MATULANE
MEKINIST
MENOPUR
MESNEX
MODERIBA 1000 DOSE PACK
MODERIBA 1200 DOSE PACK
MOZOBIL
MUSTARGEN
NATPARA
NEULASTA
NEULASTA ONPRO
NEUPOGEN
NEXAVAR
NILANDRON
NINLARO
NORDITROPIN FLEXPRO
NORTHERA
NORVIR
NPLATE
NUCALA
NUPLAZID
NUTROPIN AQ NUSPIN 10
NUTROPIN AQ NUSPIN 20
NUTROPIN AQ NUSPIN 5
NUTROPIN AQ PEN
OCALIVA
OCTREOTIDE ACETATE
ODEFSEY
OFEV
OLYSIO
OMNITROPE
OPDIVO
OPSUMIT

© 2021 Taylor EA et al. *JAMA Network Open.*

ORENCIA
ORENCIA CLICKJECT AUTOINJECTOR
ORENCIA PRE-FILLED SYRINGE
ORKAMBI
ORTHOVISC
OTEZLA
OTEZLA STARTER PACK
PEGASYS
PEGASYS PROCLICK
PLEGRIDY
PLEGRIDY PEN
PLEGRIDY PEN STARTER PACK
PLEGRIDY STARTER PACK
POMALYST
PRALUENT
PREZCOBIX
PREZISTA
PROCRIT
PROGRAF
PROMACTA
PULMOZYME
RAPAMUNE
REBIF
REBIF REBIDOSE
REBIF REBIDOSE TITRATION PACK
REGRANEX
REMICADE
REMODULIN
REPATHA
REVLIMID
REYATAZ
RIBASPHERE RIBAPAK
RIBAVIRIN
SABRIL
SAMSCA
SANCUSO
SANDOSTATIN
SANDOSTATIN LAR DEPOT
SAXENDA

© 2021 Taylor EA et al. *JAMA Network Open.*

SELZENTRY
SENSIPAR
SEROSTIM
SIMPONI
SIMPONI ARIA
SIROLIMUS
SOMATULINE DEPOT
SOMAVERT
SOVALDI
SPRIX
SPRYCEL
STELARA
STIVARGA
STRIBILD
SUBSYS
SUMAVEL DOSEPRO
SUSTIVA
SUTENT
SYLATRON
SYNVISC
SYNVISC ONE
SYPRINE
TACROLIMUS
TAFINLAR
TAGRISSO
TALTZ
TARCEVA
TARGRETIN
TASIGNA
TECFIDERA
TECFIDERA STARTER PACK
TECHNIVIE
TEMOZOLOMIDE
TETRABENAZINE
THALOMID
THYROGEN
TIVICAY
TOBI
TOBI PODHALER

© 2021 Taylor EA et al. *JAMA Network Open.*

TRACLEER
TRIUMEQ
TRULICITY
TRUVADA
TYKERB
TYSABRI
TYVASO
UPTRAVI
VALCHLOR
VALCYTE
VALGANCICLOVIR HYDROCHLORIDE
VECAMYL
VEMLIDY
VENCLEXTA
VICTOZA
VIEKIRA PAK
VIEKIRA XR
VIRACEPT
VIRAMUNE XR
VIREAD
VIVITROL
VOTRIENT
XALKORI
XELJANZ
XELJANZ XR
XELODA
XENAZINE
XGEVA
XIAFLEX
XOLAIR
XTANDI
XYREM

© 2021 Taylor EA et al. *JAMA Network Open.*
